# Pharmacotherapies for Central Post-Stroke Pain: A Systematic Review and Network Meta-Analysis

**DOI:** 10.1155/2022/3511385

**Published:** 2022-08-18

**Authors:** Zheng Bo, Yang Jian, Li Yan, Gu Gangfeng, Luo Xiaojing, Luo Xiaolan, Chen Zhao, Huang Ke, Fan Yang, Li Maoxia, Wang Jian

**Affiliations:** ^1^Department of Neurology, Ya'an People's Hospital, China; ^2^Department of Laboratory, Ya'an People's Hospital, China; ^3^North Sichuan Medical College, China

## Abstract

**Background:**

Central post-stroke pain (CPSP) is a common condition. Several pharmacotherapies have been applied in practice. However, the comparative effectiveness among these pharmacotherapies is unknown.

**Aim:**

The aim of this study is to study the comparative effectiveness among differential pharmacotherapies for CPSP through a network meta-analysis.

**Methods:**

We searched MEDLINE, EMBASE, Cochrane Central Register of Controlled Trials (CENTRAL), and Web of Science from inception to 30 March 2022, without any language restriction. Two reviewers independently screened the retrieved articles, extracted data, and evaluated the risk of bias (RoB). The outcome of interest of the study was the change in the scores of pain intensity scales. We estimated standard mean differences (SMDs) between treatments and calculated corresponding 95% CIs.

**Results:**

Thirteen randomized controlled trials (529 participants) were included after a screen of 1774 articles. Compared with placebo, pamidronate (SMD -2.43, 95% CI -3.54 to -1.31; *P* − score = 0.93), prednisone (SMD -2.38, 95% CI -3.09 to -1.67; *P* − score = 0.92), levetiracetam (SMD -2.11, 95% CI -2.97 to -1.26; *P* − score = 0.87), lamotrigine (SMD -1.39, 95% CI -2.21 to -0.58; *P* − score = 0.73), etanercept (SMD -0.92, 95% CI -1.8 to -0.03; *P* − score = 0.59), and pregabalin (SMD -0.46, 95% CI -0.71 to -0.22; *P* − score = 0.41) had significantly better treatment effect. Pamidronate, prednisone, and levetiracetam ranked as the first three most effective treatments. In subgroup analyses, prednisone, levetiracetam, lamotrigine, and pregabalin were more effective than placebo as oral pharmacotherapies, while etanercept was more effective than placebo as injectable pharmacotherapy.

**Conclusions:**

Our study confirmed that pamidronate, prednisone, and guideline-recommended anticonvulsants were effective for reducing pain intensity for CPSP. Pamidronate and prednisone showed better effect than other pharmacotherapies, which warrants further investigation.

## 1. Introduction

Central post-stroke pain (CPSP) is a type of chronic neuropathic pain caused by lesions to the central somatosensory nervous system, as stated by the International Association for the Study of Pain (IASP). CPSP is a common but long-neglected condition after stroke, and it therefore is still an underappreciated sequela of stroke, which impairs quality of life, interrupts rehabilitation or lengthens the period of rehabilitation, lowers the sleep quality, causes mood disturbances, and increases the risk of suicide [1–7]. The prevalence of CPSP varies across studies. The largest sample size study, recruiting 15,754 participants with ischemic, reported that 2.7% of the participants developed CPSP one year after stroke [8]. A recent systematic review reported that the pooled prevalence of CPSP in patients with stroke was 11%, and 31% of the patients developed CPSP within one month after stroke onset [9]. The high prevalence of CPSP and the heavy disease burden warrant studies that focus on effective treatments for it.

Pharmacotherapies are still the first-line treatment for CPSP. In the recent 20 years, several classes of medications for CPSP are recommended, such as anticonvulsants, antidepressants, opioids, analgesics, and steroids [1, 2, 4–6]. The IASP has published a systematic review [10], recommending tricyclic antidepressants, gabapentin, pregabalin, and serotonin noradrenaline reuptake inhibitors as first-line drugs for the treatment of CPSP. However, another systematic review argued that the recommendation for tricyclic antidepressants was based on one trial of 15 participants, and the tricyclic antidepressants were found with similar effects to placebo, which were therefore should not be recommended for the treatment of CPSP [5]. Several randomized controlled trials (RCTs) emerged after these two systematic reviews, proposing new pharmacological treatments for CPSP [11–13]. The aforementioned studies raised an important and clinically relevant question for clinicians and patients, which is the relatively better treatment for CPSP? Regarding that few head-to-head trials have been conducted to compare the effectiveness of differential pharmacotherapies for CPSP, we aimed to perform a network meta-analysis based on a systematic review to answer the question.

We conducted a systematic review and network meta-analysis, including RCTs that recruited patients with CPSP, assessed the efficacy of pharmacological treatments in the reduction of pain intensity of CPSP, and provided the evidence of comparative effectiveness among the differential pharmacotherapies.

## 2. Methods

The systematic review was conducted according to the Preferred Reporting Items for Systematic Reviews and Meta-Analysis (PRISMA) statement and PRISMA for network meta-analysis [14]. The systematic review and meta-analysis did not include participant-level data, so ethical approval for the study was not required.

### 2.1. Study Source

Ovid MEDLINE, EMBASE, the Cochrane Controlled Register of Trials (CENTRAL) and Web of Science were searched from inception to 30 March 2022, without any language restriction. We searched the databases with the search strategies combining keywords or medical subject headings of study design, participants, interventions, and controls. We also searched previously published systematic reviews and meta-analyses and read the reference lists of the reviews to examine whether there were missing studies. In addition, clinical registries (https://www.clinicaltrials.gov and http://www.chictr.org.cn) were investigated for potentially eligible studies. The search strategy is shown in the Supplement 1.

### 2.2. Selection Criteria

The inclusion criteria included are as follows: (1) with randomized controlled trial (RCT) design; (2) adult participants who had ischemic stroke or intracranial hemorrhage and had CPSP after stroke (we did not limit the diagnostic criteria and the onset time periods for CPSP, since the diagnostic criteria were heterogeneous across trials and many participating physicians diagnosed patients according to practice experience. In addition, the onset time was seldom reported in the included trials); (3) RCTs that tested the effectiveness of any of the following interventions: antiepileptic drugs (e.g., lamotrigine, gabapentin, carbamazepine, and levetiracetam), analgesic drugs (e.g., lidocaine, morphine), steroids (e.g., prednisolone), antidepressants (e.g., amitriptyline), etanercept, and pamidronate, naloxone; the control interventions included usual care, or placebo; and (4) RCTs that assessed pain intensity and adverse events.

The exclusion criteria included are as follows: (1) RCTs with insufficient data for analysis (i.e., the studies that did not provide means, standard deviations, number of events, or the number of participants in each arm; the studies that focused on the topic but did not assess pain intensity) and (2) RCTs that were reported in the form of conference abstracts, research letters, or news reports.

The retrieved papers were screened independently by two reviewers. They first read the title and abstracts of the articles and made the first decision, and they obtained the full-text copies for further evaluation when decisions could not make upon titles and abstracts. Discrepancies between the two reviewers were solved by group discussion and arbitrated by a third reviewer.

### 2.3. Outcome Measurement

The outcome of interest of the study was the change in the scores of pain intensity scales. The assessments of pain intensity included visual analog scale (VAS), numeric rating scales (NRS), or Likert scales. The VAS score was rated by asking a participant to rate in a 100-mm line (with 0 mm indicating no pain and 100 mm indicating the worst pain). The NRS score was rated similarly to the VAS scale, with the rating 0 indicating no pain and the rating number 10 indicating the most severe pain. The Likert scales were the assessments of pain intensity with 4, 5, or 7 points to classify the severity of pain, and a larger number of the score signals a more severe pain.

### 2.4. Data Extraction

We used standardized electronic forms to extract data from the included RCTs. Two reviewers independently extracted the data. They extracted the characteristics of the included RCTs, baseline parameters of the included participants, details of the interventions and controls, and outcome measures. The characteristics of the included RCTs included the name of the first author, the year of publication, the total sample size, and the study design (parallel or cross-over). The baseline characteristics of the participants included the mean age, the sex, and the cause of CPSP. The dose and usage (oral administration, intravenous administration, or other methods) were recorded for each RCT, and the types of control, placebo or usual care, were also recorded. We collected the names of the pain intensity scales and the assessment time. A third reviewer checked and validated the extracted data and passed the clear data to a statistician.

### 2.5. Risk of Bias Assessment

The risk of bias (RoB) of the included RCTs was assessed by using the second version of the Cochrane risk of bias (RoB 2.0), which contains five domains for assessment. Under each domain, there are several signal questions required to be answered. In the assessment of the missing values domain, we tried to contact the corresponding authors of trials with missing values to help make a judgment of RoB. The answers to these questions contribute to judgments of the RoB for a specific study—low, some concerns, or high RoB. The RoB 2.0 provides an overall rating of a study, for which we could classify the quality of the included RCTs into low, some concerns, or high RoB.

### 2.6. Data Synthesis

We estimated the effect size of each pharmacotherapy against the control using a traditional meta-analysis of head-to-head comparisons (direct comparison evidence) and calculated the corresponding 95% CIs. Regarding that pain intensity was assessed with different scales (100-VAS, 10-NRS, and other scales) in the included RCTs, we calculated the effect size using standard mean difference (SMD). We performed a frequentist-approach network meta-analysis [15], comparing differential pharmacotherapies in the reduction of pain intensity. The network meta-analysis was conducted using a random-effect model. We first estimated the effect size of each pharmacotherapy against placebo and calculated the corresponding 95% CIs. Secondly, we performed pairwise comparisons among differential pharmacotherapies and presented their effects relative to each other in a league table. After computing the effect size of each pharmacotherapy relative to placebo, we calculated the probability of each pharmacotherapy becoming the best treatment through the mean probability score (*P*-score). We drew a forest plot showing the SMD for each pharmacotherapy against placebo along with corresponding *P*-scores. In a network meta-analysis, a *p* value for the determination of significant difference between two treatments is not provided, which is determined by whether a 95% CI of the SMD crossed the null value line, indicating no significant difference between the two treatments [16]. We classified the size of SMD into small, moderate, and large with cut-off points of 0.2, 0.5, and 0.8, respectively; we adopted the moderate size (SMD = 0.5) as a clinically relevant importance difference [17].

We examined the consistency of the network meta-analysis by comparing the estimates from direct, indirect, and network estimates, and a *p* < 0.05 from the *z*-test for these estimates would be viewed as a sign of inconsistency. The heterogeneity of the network meta-analysis was checked by using global *I*^2^ statistics and tau-squared value when an *I*^2^> 50% or a tau-squared value >0.36 was considered a sign of large heterogeneity. The design-by-treatment analysis would be performed when there was large heterogeneity, aiming to detect the source of heterogeneity.

We performed several subgroup analyses to identify whether the treatment effect differs in different subgroups. Regarding that the estimation method of heterogeneity is different between network meta-analysis and traditional meta-analysis, we performed subgroup analyses even though small global heterogeneity was found in the analysis. We classified the study design as cross-over versus parallel, the subtype of condition as stroke versus stroke or spinal cord injury, the administration method of intravenous versus oral, and the measurement method of the 100-mm VAS scale versus other scales. The first subgroup analysis was to check whether the study design affected the results. The second subgroup analysis was conducted because four included studies recruited patients with CPSP caused by stroke or spinal cord injury, while the other studies included only patients with stroke. The third subgroup analysis was to test whether the administration method affected the study results since a previous study demonstrated that placebos with invasive administration had larger placebo effects than placebo pills [18]. The fourth subgroup analysis was to clarify whether the studies that used the 100-mm VAS scale had different results when compared with studies that used other scales. The fifth subgroup analysis was to clarify whether the treatment duration had an impact on the study results. We classified the treatment duration into short term (<6 weeks), medium term (6-12 weeks), and long term (>12 weeks) and reperformed the analysis. All the analyses were performed by using R 4.0.1 (https://www.r-project.org/) with the netmeta package.

## 3. Results

### 3.1. Characteristics of the Included RCTs

**Figure 1:**
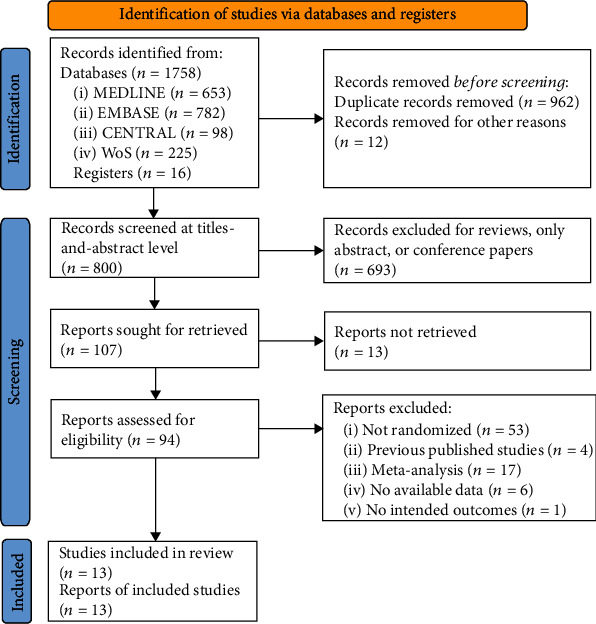
Screening process. Abbreviations: CENTRAL: Cochrane Central Register of Controlled Trials. WoS: Web of Science. Footnotes: In records removed for other reasons, the reasons included ongoing trials with only register numbers (*n* = 8), terminated trials with only notice (*n* = 3), and one article without authors and publication source. “no available data” refers to the studies reported the assessment of pain intensity or adverse events but has no relevant data to extract for analysis. “no intended outcomes” refers to the studies reported no assessment of pain intensity or adverse events.

After the systematic search, we retrieved 1758 records from electronic databases and 16 records from clinical registers. Thirteen RCTs were finally included [11–13, 19–28], after we excluded duplicate records (*n* = 962), records reporting as reviews, abstracts only, or conference papers (*n* = 693), reports that were not retrieved (*n* = 13), studies that were not randomized design (*n* = 53), studies that were previously published (*n* = 4), meta-analyses (*n* = 17), studies with unavailable data (*n* = 6), without intended outcomes (*n* = 1), and other reasons (*n* = 12). Detailed results of the screening process are shown in Figure 1.

Table 1 shows the characteristics of the included RCTs. The included 13 RCTs had included 572 participants, and these RCTs were published from the year 1992 to 2020. Nine RCTs included only patients with post-stroke pain, and 4 RCTs recruited patients with post-stroke pain and neuropathic pain owing to spinal cord injury or other central nervous lesions. Seven RCTs adopted parallel design, and 6 RCTs adopted cross-over design. The assessed pharmacotherapies included amitriptyline, carbamazepine, etanercept, ketamine, lamotrigine, levetiracetam, lidocaine, morphine, naloxone, pamidronate, prednisolone, and pregabalin. Seven RCTs assessed oral pharmacotherapies, four assessed intravenous pharmacotherapies, one assessed a peri-spinal injection, and one assessed a self-powered disposable patch. Seven RCTs applied the 100-VAS scale to assess pain intensity, three applied the 10-NRS, one used both the 100-VAS and 10-NRS, one used a 10-step verbal scale, and one used the other scale.

### 3.2. Risk of Bias Assessment

**Figure 2:**
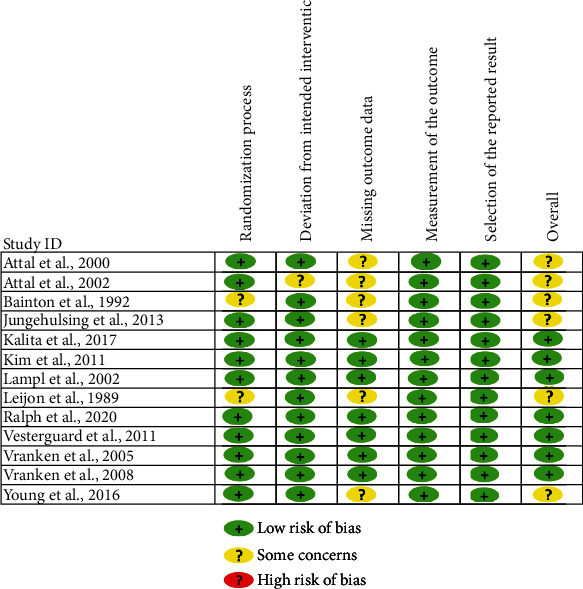
Risk of bias assessment. Footnotes: The risk of bias was assessed by using the revised Cochrane Risk of Bias tool (RoB 2.0). Five domains of each RCT were assessed and classified into low RoB, some concerns, or high RoB, and an overall assessment was therefore provided after the assessment of the five domains.

Figure 2 shows the results of the RoB assessment. Seven RCTs were classified with a low risk of bias, and the rest 6 RCTs were classified with some concerns. The most rated some-concerns domain was in missing outcome data, which involved 6 RCTs.

The second most rated some-concerns domain was the randomization process, which involved 2 RCTs.

### 3.3. The Network Meta-Analysis of Pharmacotherapies for Pain Intensity

**Figure 3:**
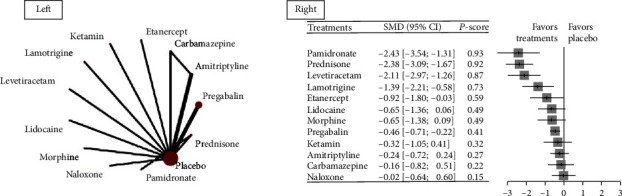
Network graph and forest plot for the change in pain intensity. Abbreviations: SMD: standard mean difference. Footnotes: The left side of the figure shows the net graph of the network meta-analysis comparing differential pharmacotherapies in the change in pain intensity after treatment. The right side shows the forest plots comparing differential pharmacotherapies against placebo, and the treatments were ranked by *P*-scores—the mean probability of a treatment becoming the best one. The treatment at the top was the one with the highest *P*-score.

A network meta-analysis comparing differential pharmacotherapies in reducing pain intensity was conducted, which included 13 RCTs and 572 participants.  Figure 3 shows the results of a network meta-analysis comparing each pharmacotherapy against placebo, which includes the effect sizes and *P*-scores.  Table 2 shows the pairwise comparison between any of the two pharmacotherapies.

The analysis showed that, compared with placebo, pamidronate (SMD -2.43, 95% CI -3.54 to -1.31), prednisone (SMD -2.38, 95% CI -3.09 to -1.67), levetiracetam (SMD -2.11, 95% CI -2.97 to -1.26), lamotrigine (SMD -1.39, 95% CI -2.21 to -0.58), etanercept (SMD -0.92, 95% CI -1.8 to -0.03), and pregabalin (SMD -0.46, 95% CI -0.71 to -0.22) had significantly better treatment effect. Pamidronate, prednisone, and levetiracetam ranked the first three most effective treatments (*P*-scores were 0.93, 0.92, and 0.87, respectively). Pamidronate, prednisone, levetiracetam, lamotrigine, and etanercept showed a moderate size of improvement when compared with placebo, which was regarded as a clinically relevant importance difference.

In pairwise comparisons, we found that pamidronate caused significantly lower scores of pain intensity than carbamazepine (SMD -2.27, 95% CI -3.57 to -0.97), etanercept (SMD -1.51, 95% CI -2.93 to -0.09), ketamin (SMD -2.11, 95% CI -3.43 to -0.78), lidocaine (SMD -1.78, 95% CI -3.1 to -0.46), morphine (SMD -1.78, 95% CI -3.12 to -0.45), naloxone (SMD -2.4, 95% CI -3.68 to -1.13), and pregabalin (SMD -1.96, 95% CI -3.1 to -0.82). In addition, we found that prednisone was significantly better than amitriptyline, carbamazepine, etanercept, ketamine, lidocaine, morphine, and naloxone, and we also found that levetiracetam was significantly better than amitriptyline, carbamazepine, ketamine, lidocaine, morphine, and naloxone. More details are shown in Table 2.

The network meta-analysis showed no heterogeneity (global *I*^2^ value = 0%, tau − squared value = 0, *p* value = 0.957). The consistency test showed no evidence of inconsistency between direct and indirect comparison.

### 3.4. Subgroup Analyses

The results of the subgroup analyses were shown in the supplementary files.

#### 3.4.1. Oral Administration versus Other Administrations

Six RCTs assessed oral pharmacotherapies in the treatment of CPSP. The network meta-analysis showed that oral prednisone (SMD -2.38, 95% CI -3.09 to -1.67; *P* − score = 0.94), levetiracetam (SMD -2.11, 95% CI -2.97 to -1.26; *P* − score = 0.87), and lamotrigine (SMD -1.39, 95% CI -2.21 to -0.58; *P* − score = 0.69) were the three most effective treatments. The SMDs were calculated using the placebo as a reference control.

Seven RCTs assessed pharmacotherapies with other forms of administration. The network meta-analysis showed that etanercept (SMD -0.92, 95% CI -1.8 to -0.03; *P* − score = 0.82) was the most effective.

#### 3.4.2. Study Design

Six RCTs adopted cross-over design. The network meta-analysis showed that levetiracetam (SMD -2.11, 95% CI -2.97 to -1.26; *P* − score = 0.98) and lamotrigine (SMD -1.39, 95% CI -2.21 to -0.58; *P* − score = 0.84) were the most effective pharmacotherapies.

Seven RCTs applied parallel design. The network meta-analysis showed that pamidronate (SMD -2.43, 95% CI -3.66 to -1.2; *P* − score = 0.92), prednisone (SMD -2.38, 95% CI -3.18 to -1.58; *P* − score = 0.91), and pregabalin (SMD -0.52, 95% CI -0.91 to -0.13; *P* − score = 0.44) were the three most effective pharmacotherapies.

#### 3.4.3. The Causes of CPSP

Nine RCTs included CPSP patients caused by merely stroke. The network meta-analysis showed that pamidronate (SMD -2.43, 95% CI -3.54 to -1.31; *P* − score = 0.9), prednisone (SMD -2.38, 95% CI -3.09 to -1.67; *P* − score = 0.9), and levetiracetam (SMD -2.11, 95% CI -2.97 to -1.26; *P* − score = 0.83) were the three most effective treatments.

Four RCTs recruited CPSP patients caused by stroke, spinal cord injury, or other central nervous lesions. The network meta-analysis showed that pregabalin (SMD -0.85, 95% CI -1.49 to -0.2; *P* − score = 0.79) was the most effective.

#### 3.4.4. Measure Methods

Seven RCTs applied a 100-mm VAS scale to measure CPSP. The network meta-analysis showed that pregabalin (mean difference -14, 95% CI –24.27 to -3.73; P-score = 0.82) was the most effective treatment.

The other six RCTs adopted 10-NRS or other scales to measure CPSP. The network meta-analysis showed that levetiracetam (SMD -2.11, 95% CI -2.97 to -1.26; *P* − score = 0.98), lamotrigine (SMD -1.39, 95% CI -2.21 to -0.58; *P* − score = 0.81), and etanercept (SMD -0.92, 95% CI -1.8 to -0.03; *P* − score = 0.65) were the three most effective treatments.

#### 3.4.5. Treatment Duration

Nine RCTs included pharmacotherapies with short-term treatment duration. The network meta-analysis showed that pamidronate was the most effective treatment (SMD -2.43, 95% CI -3.54 to -1.31; *P* − score = 0.95).

Three RCTs included pharmacotherapies with medium-term duration. The network meta-analysis showed that levetiracetam was the most effective treatment (SMD -2.11, 95% CI -2.97 to -1.26; *P* − score = 0.96).

One RCT assessed the long-term (52 weeks) effect of amitriptyline, and the result showed slight, but not significant difference between amitriptyline and placebo (SMD -0.25, 95% CI -0.89 to 0.39, *p* value = 0.443).

More details about the results of the subgroup analyses are shown in the Supplement 2.

### 3.5. Safety

The adverse events were diversely described in the included RCTs, and the network meta-analysis was therefore not performed. No serious adverse event was reported for the included pharmacotherapies. One out of 13 patients receiving etanercept reported shingles [13]. Among the 11 patients taking pamidronate, two reported fewer, one reported myalgia, and one reported infusion site reactions [11]. Three out of the 21 patients receiving levetiracetam withdrew from the trial because of adverse events [22], while 9 out of 110 patients receiving gabapentin did [23]. Two of the 18 patients receiving amitriptyline reported moderate events that needed a reduction in dosage [24]. Six of the 15 patients receiving morphine reported somnolence or nausea [20]. Six of the 14 patients receiving lamotrigine reported mild rash or severe headache [26]. In general, all the reported adverse events were mild to moderate and needed no special medical treatment.

## 4. Discussion

Our study aimed to compare differential pharmacotherapies for the treatment of CPSP, a common condition after stroke, through a network meta-analysis. The results of the meta-analysis showed that several pharmacotherapies, including pamidronate, prednisone, levetiracetam, lamotrigine, etanercept, and pregabalin, were significantly superior over placebo in the reduction of CPSP pain intensity. Pamidronate and prednisone showed significantly better treatment effects than most of the other pharmacotherapies. We performed several subgroup analyses, and we found that prednisone, levetiracetam, lamotrigine, and pregabalin were more effective than placebo as oral pharmacotherapies, while etanercept was more effective than placebo as injectable pharmacotherapy. For the cause of CPSP, pregabalin was the most effective when patients with stroke, spinal cord injury, or other types of central nervous lesions were all included. For the reduction of 100-VAS scores, pregabalin showed a better effect than pamidronate, prednisone, naloxone, and placebo. For the reduction of other pain scales, levetiracetam and lamotrigine seemed to be better than pregabalin, amitriptyline, carbamazepine, and placebo. All pharmacotherapies caused no serious adverse events, although different types of adverse events and event rates were reported across the included RCTs. We classified the treatment duration into short, medium, and long term, and this subgroup analysis showed that pamidronate was the most effective in pharmacotherapies with short-term duration and levetiracetam was the most effective in pharmacotherapies with medium-term duration. No serious adverse event was reported for the included pharmacotherapies, which might indicate that current pharmacotherapies were safe.

To the best of our knowledge, this was the first network meta-analysis that was performed to search for a relatively better pharmacotherapy for CPSP. Several of our findings were consistent with previous systematic reviews. First, we found that several anticonvulsants were effective in reducing pain intensity for CPSP, such as levetiracetam, lamotrigine, and pregabalin. Among these anticonvulsants, pregabalin was previously recommended by the IASP systematic review [10]. The effectiveness of pregabalin was mainly supported by an RCT from South Korea recruiting 219 participants, which was the largest sample size RCT in this meta-analysis. Our meta-analysis added new finding that levetiracetam and lamotrigine seemed to have larger treatment effect than pregabalin, although the finding was mainly supported by two small sample size studies [12, 22], and after we limited the analysis to participants taking oral administration, the results were consistent. The finding informed that RCTs of larger size might be warranted to examine whether levetiracetam and lamotrigine could be recommended as treatment options for CPSP. Second, amitriptyline was ineffective when compared with placebo, as reported in a previous systematic review [5]. Our study confirmed this finding, and we found that amitriptyline was inferior to anticonvulsants (e.g., levetiracetam and lamotrigine). The recommendation for the use of amitriptyline in CPSP management should be reevaluated. Third, we found that lidocaine and morphine were not effective in the management of CPSP, since the 95%CIs of their SMDs included the null value when compared with placebo, which indicates the insignificant difference between them and placebo. The finding was consistent with a recently published systematic review [29].

We had several new findings that needed further investigations. First, pamidronate was superior to placebo and relatively more effective than pregabalin. Pamidronate is normally used to treat high blood calcium levels and certain bone problems [30], the mechanism of how it works should be clearly demonstrated before practice recommendation. In addition, the effect of pamidronate was supported by a small size RCT, which needed further confirmation to rule out the possibility of the small study effect [31]. Second, etanercept, an agent of anti-inflammatory tumor necrosis factor inhibitors (anti-TNF), normally used to treat certain types of arthritis [32], was found effective in the treatment of CPSP. This finding might infer that anti-inflammatory pharmacotherapy had a promising future in the treatment of CPSP, and the causes of CPSP pain might involve the anti-inflammatory pathways. Third, all the effective pharmacotherapies had only short-term benefits. The evidence of long-term effect (>3 months) is lacking. This might raise concerns about the safety and tolerance of the long-term use of the pharmacotherapies for CPSP. In summary, future studies might focus on the evaluation of the effect of pamidronate and etanercept, the mechanism of how these drugs take effect, and the long-term effect and safety of the included pharmacotherapies.

Our study has several limitations. First, like the other systematic reviews and meta-analyses, our study might have also missed some of the eligible RCTs, although we adopted comprehensive search strategies in the electronic databases. Second, the baseline characteristics might vary enormously across different studies, which might cause bias in the network meta-analysis. For example, some RCTs might include participants who had failed first-line pharmacotherapies. However, owing to the incompleteness of information disclosure, a meta-regression or subgroup analysis was unable to perform because of the lack of relevant information. In future studies, acquiring individual participant-level data might solve the problem. Third, cost-effectiveness was not assessed in our study, since there was a lack of relevant data. Health economic evaluation is necessary for clinicians and patients. When two pharmacotherapies (e.g., levetiracetam and lamotrigine) had similar treatment effects, the one with lower cost might be preferred. In addition, owing to the lack of outcomes other than pain intensity in the included studies, the outcomes—functional status and quality of life—were not assessed in our meta-analysis. Fourth, we did not assess the certainty of evidence by using the GRADE approach, because we assumed that the GRADE approach is not suitable for this network meta-analysis, because the network meta-analysis contains many pairs of indirect comparisons which will be downregulated by the GRADE approach. Additionally, the network meta-analysis focused more on the probability of a treatment being the best one, so we provided the *P*-scores, a measurement of the probability of a treatment being the best out of several treatment options, to help the readers to understand the credibility of the evidence.

In summary, our network meta-analysis confirmed that anticonvulsants were effective in the management of CPSP, as they were recommended in practice guidelines [33, 34]. The other promising treatments (pamidronate, levetiracetam, lamotrigine, and etanercept) should be examined in future RCTs. The long-term effectiveness, safety, and cost-effectiveness of these pharmacotherapies should also be further evaluated.

## Figures and Tables

**Table 1:** Characteristics of the included trials.

Study ID	Sample size	Cause of pain	RCT design	Intervention	Administration method of the intervention and treatment duration	Control	Pain scale	Assessment time
[19]	16	Stroke or spinal cord injury	Cross-over, 3-week washout	Lidocaine, 5 mg/kg/day	IV; 3 weeks	0.9% saline	100-VAS	Until post-infusion
[20]	16	Stroke or spinal cord injury	Cross-over, 3-week washout	Morphine (9-30 mg/day; 16 ± 6.1 mg per day in average)	IV; 3 weeks	0.9% saline	100-VAS	Until post-infusion
[21]	20	Stroke	Cross-over, 2-week washout	Naloxone, 8 mg/session	IV; 2 weeks	0.9% saline	100-VAS	Until post-infusion
[22]	42	Stroke	Cross-over, 2-week washout	Levetiracetam, started at 1000 mg and increased every 2 weeks to 3000 mg	Oral; 8 weeks	Placebo pills	10-NRS	Post-treatment
[12]	58	Stroke	Parallel	Prednisolone, 10 mg/day for 2 months	Oral; 4 weeks	No prednisolone	100-VAS	Post-treatment
[23]	219	Stroke	Parallel	Pregabalin, 150-600 mg/day	Oral; 12 weeks	Placebo pills	10-NRS	Post-treatment
[24]	39	Acute thalamic stroke	Parallel	Amitriptyline, 10-75 mg	Oral; 52 weeks	Placebo pills	NA	Censor point within one year
[25]	15	Stroke	Cross-over, 1-week washout	Amitriptyline, 12.5-50 mg	Oral; 4 weeks	Carbamazepine (200-800 mg) or placebo pills	10-step verbal scale	Post-treatment
[13]	26	Ischemic stroke in MCA territory; ICH stroke in basal ganglia	Parallel	Etanercept, 25 mg	Peri-spinal; 2 weeks	0.9% saline	100-VAS or 10-NRS	Post-treatment
Vesterguard et al., 2001 [26]	27	Stroke	Cross-over, 2-week washout	Lamotrigine, tapered from 25 mg/day to 200 mg/d	Oral; 8 weeks	Placebo pills	10-NRS	Post-treatment
Eun Young et al., 2016 [11]	21	First-ever stroke	Parallel	Pamidronate, 60 mg/session, 3 infusions every two days for six days	IV; 2 weeks	Oral prednisolone 1 mg/kg/day	100-VAS	Post-treatment
[27]	33	Stroke, spinal cord lesion or other central nervous lesions	Parallel	Ketamine 50-75 mg/session, 5 sessions	Self-powered disposable patch; one week	Placebo patch	100-VAS	Post-treatment
[28]	40	Stroke, spinal cord lesion or other central nervous lesions	Parallel	Pregabalin, 150-600 mg/day, 4 weeks	Oral; 4 weeks	Placebo pills	100-VAS	Post-treatment

**Table 2:** Pairwise comparisons of treatments.

Amitriptyline	-0.07 (-0.79; 0.64)	.	.	.	.	.	.	.	.	-0.24 (-0.72; 0.24)	.	.
-0.08 (-0.75; 0.58)	Carbamazepine	.	.	.	.	.	.	.	.	-0.15 (-0.87; 0.57)	.	.
0.68 (-0.33; 1.68)	0.76 (-0.35; 1.86)	Etanercept	.	.	.	.	.	.	.	-0.92 (-1.80; -0.03)	.	.
0.08 (-0.79; 0.95)	0.17 (-0.82; 1.15)	-0.59 (-1.74; 0.55)	Ketamin	.	.	.	.	.	.	-0.32 (-1.05; 0.41)	.	.
1.15 (0.21; 2.10)	1.24 (0.18; 2.29)	0.48 (-0.72; 1.68)	1.07 (-0.02; 2.16)	Lamotrigine	.	.	.	.	.	-1.39 (-2.21; -0.58)	.	.
1.88 (0.89; 2.86)	1.96 (0.87; 3.04)	1.20 (-0.03; 2.43)	1.79 (0.67; 2.92)	0.72 (-0.46; 1.90)	Levetiracetam	.	.	.	.	-2.11 (-2.97; -1.26)	.	.
0.41 (-0.45; 1.27)	0.49 (-0.48; 1.46)	-0.27 (-1.40; 0.86)	0.33 (-0.69; 1.34)	-0.75 (-1.83; 0.34)	-1.47 (-2.58; -0.35)	Lidocaine	.	.	.	-0.65 (-1.36; 0.06)	.	.
0.41 (-0.47; 1.28)	0.49 (-0.50; 1.48)	-0.27 (-1.42; 0.88)	0.32 (-0.71; 1.36)	-0.75 (-1.84; 0.35)	-1.47 (-2.60; -0.34)	-0.00 (-1.02; 1.02)	Morphine	.	.	-0.65 (-1.38; 0.09)	.	.
-0.22 (-1.00; 0.57)	-0.13 (-1.04; 0.77)	-0.89 (-1.97; 0.19)	-0.30 (-1.26; 0.66)	-1.37 (-2.39; -0.35)	-2.09 (-3.15; -1.03)	-0.62 (-1.57; 0.32)	-0.62 (-1.58; 0.34)	Naloxone	.	-0.02 (-0.64; 0.60)	.	.
2.19 (0.98; 3.40)	2.27 (0.97; 3.57)	1.51 (0.09; 2.93)	2.11 (0.78; 3.43)	1.03 (-0.34; 2.41)	0.31 (-1.09; 1.72)	1.78 (0.46; 3.10)	1.78 (0.45; 3.12)	2.40 (1.13; 3.68)	Pamidronate	.	-0.05 (-0.91; 0.81)	.
-0.24 (-0.72; 0.24)	-0.16 (-0.82; 0.51)	-0.92 (-1.80; -0.03)	-0.32 (-1.05; 0.41)	-1.39 (-2.21; -0.58)	-2.11 (-2.97; -1.26)	-0.65 (-1.36; 0.06)	-0.65 (-1.38; 0.09)	-0.02 (-0.64; 0.60)	-2.43 (-3.54; -1.31)	Placebo	2.38 (1.67; 3.09)	0.46 (0.22; 0.71)
2.14 (1.28; 3.00)	2.22 (1.25; 3.19)	1.46 (0.33; 2.60)	2.06 (1.04; 3.07)	0.99 (-0.10; 2.07)	0.26 (-0.85; 1.38)	1.73 (0.73; 2.74)	1.73 (0.71; 2.75)	2.36 (1.41; 3.30)	-0.05 (-0.91; 0.81)	2.38 (1.67; 3.09)	Prednisone	.
0.23 (-0.31; 0.76)	0.31 (-0.40; 1.02)	-0.45 (-1.37; 0.46)	0.14 (-0.63; 0.91)	-0.93 (-1.78; -0.08)	-1.65 (-2.54; -0.76)	-0.18 (-0.94; 0.57)	-0.18 (-0.96; 0.59)	0.44 (-0.23; 1.11)	-1.96 (-3.10; -0.82)	0.46 (0.22; 0.71)	-1.91 (-2.67; -1.16)	Pregabalin

Footnotes: The comparisons between any two treatments should be read from left to right, and the comparison estimate (expressed as standard mean difference [SMD] and its related 95%CI) is in the cell between the column-defining treatment and the row-defining treatment. The top half of the table presents SMDs from direct comparison evidence, while the bottom half of the table presents SMDs from network meta-analysis. In top half, SMDs >0 favor row-defining treatments vs. column-defining treatments. In the bottom half, SMDs <0 favor column-defining treatments. Empty cells indicate no direct comparison between two treatments.

## Supplementary Materials

Supplementary Materials Supplement 1. Search strategy. Supplement 2. Subgroup analyses.
