# Supplementary material for: Pharmacotherapies for Central Post-Stroke Pain: A Systematic Review and Network Meta-Analysis
Source: Oxid Med Cell Longev. 2022 Aug 18;2022:3511385. doi: 10.1155/2022/3511385 (PMC9410833; doi:10.1155/2022/3511385)
Supplement: Supplementary Materials — Supplement 1. Search strategy. Supplement 2. Subgroup analyses. [file 3511385.f1.docx]

**Supplement 1. Search strategy**

1. OVID MEDLINE

1. randomised controlled trial.pt.

2. randomized controlled trial.pt.

3. controlled clinical trial.pt.

4. randomized.ab.

5. randomised.ab.

6. randomly.ab.

7. or/1-6

8. limit 7 to humans

9. exp Neuralgia/

10. exp Neuropathic Pain/

11. exp Complex Regional Pain Syndromes/

12. poststroke pain. ti,ab.

13. central pain. ti,ab.

14. or/9-13

15. lidocaine. ti,ab.

16. morphine. ti,ab.

17. naloxone. ti,ab.

18. anticonvulsant. ti,ab.

19. glucocorticoid. ti,ab.

20. levetiracetam. ti,ab.

21. prednisolone. ti,ab.

22. pregabalin. ti,ab.

23. amitriptyline. ti,ab.

24. lamotrigine. ti,ab.

25. ketamine. ti,ab.

26. or/15-25

45. 7 AND 14 AND 26

2. EMBASE

1. ‘randomized Controlled Trial’/exp

2. ‘randomized Controlled Trials as Topic’/exp

3. ‘randomized controlled trial’:ab,ti

4. ‘controlled clinical trial’/exp

5. ‘controlled clinical trial’:ab,ti

6. (#1 OR #2 OR #3 OR #4 OR #5) AND [humans]/lim

7. ‘Neuralgia’/exp

8. ‘Neuropathic Pain’/exp

9. ‘Complex Regional Pain Syndromes’/exp

10. ‘poststroke pain’:ab,ti

11. ‘central pain’:ab,ti

12. #7 OR #8 OR #9 OR #10 OR #11

13. ‘lidocaine’:ab,ti

14. ‘morphine’:ab,ti

15. ‘naloxone’:ab,ti

16. ‘anticonvulsant’:ab,ti

17. ‘glucocorticoid’:ab,ti

18. ‘levetiracetam’:ab,ti

19. ‘prednisolone’:ab,ti

20. ‘pregabalin’:ab,ti

21. ‘amitriptyline’:ab,ti

22. ‘lamotrigine’:ab,ti

23. ‘ketamine’:ab,ti

24. #13 OR #14 OR #15 OR #16 OR #17 OR #18 OR #19 OR #20 #21 OR #22 OR #23

41. #6 AND #12 AND #24

3. Cochrane library

1. (randomised controlled trial):ti,ab,kw

2. (poststroke pain):ti,ab,kw

3. (central pain) :ti,ab,kw

4. (Complex Regional Pain Syndromes):ti,ab,kw

5. #2 or #3 or #4

6. (lidocaine):ti,ab,kw

7. (morphine):ti,ab,kw

8. (naloxone):ti,ab,kw

9. (anticonvulsant):ti,ab,kw

10. (glucocorticoid):ti,ab,kw

11. (levetiracetam) :ti,ab,kw

12. (prednisolone):ti,ab,kw

13. (pregabalin):ti,ab,kw

14. (amitriptyline):ti,ab,kw

15. (lamotrigine):ti,ab,kw

16. (ketamine):ti,ab,kw

17. #6 or #7 or #8 or #9 or #10 or #11 or #12 or #13 or #14 or #15 or #16

28. #1 and #5 and #17

**Supplement 2. Subgroup analyses**

1. **Administration methods**

**1.1 Oral administration**


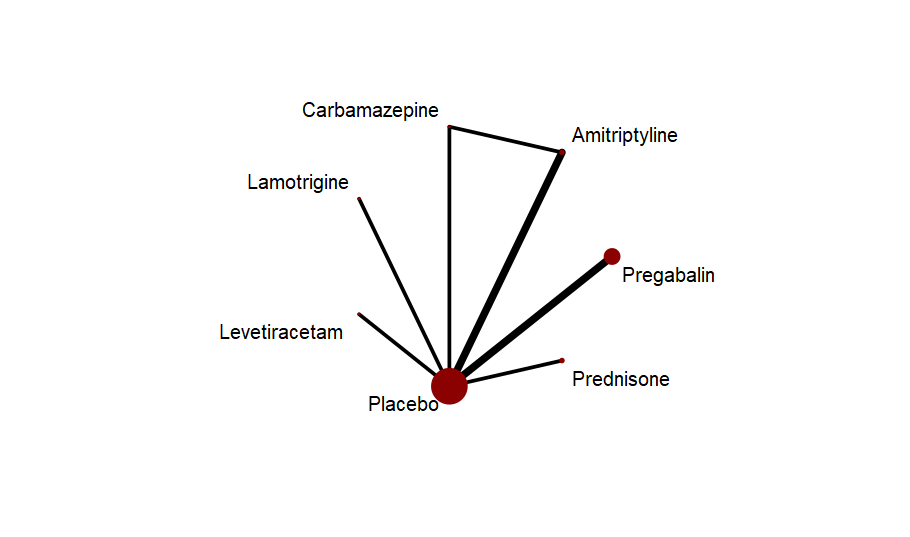


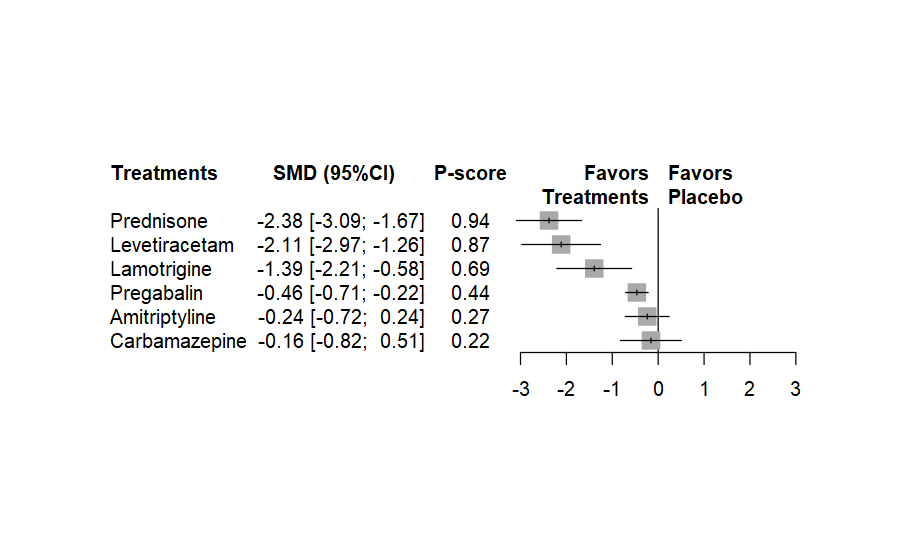


The analysis included trials assessing oral pharmacotherapies. The first-half was a netgraph, and the second-half was a forest plot showing the effect size of each pharmacotherapy against placebo and the P-score.

**1.2 Other administration methods**


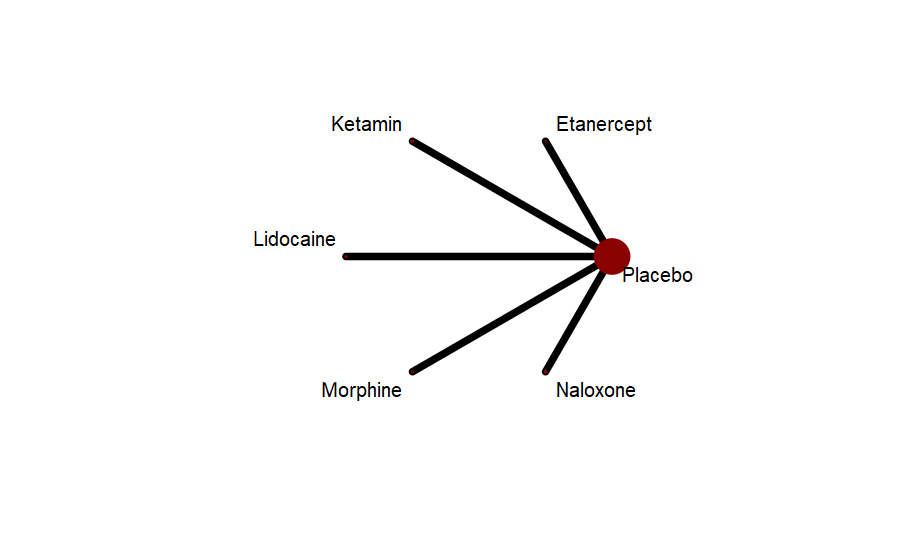


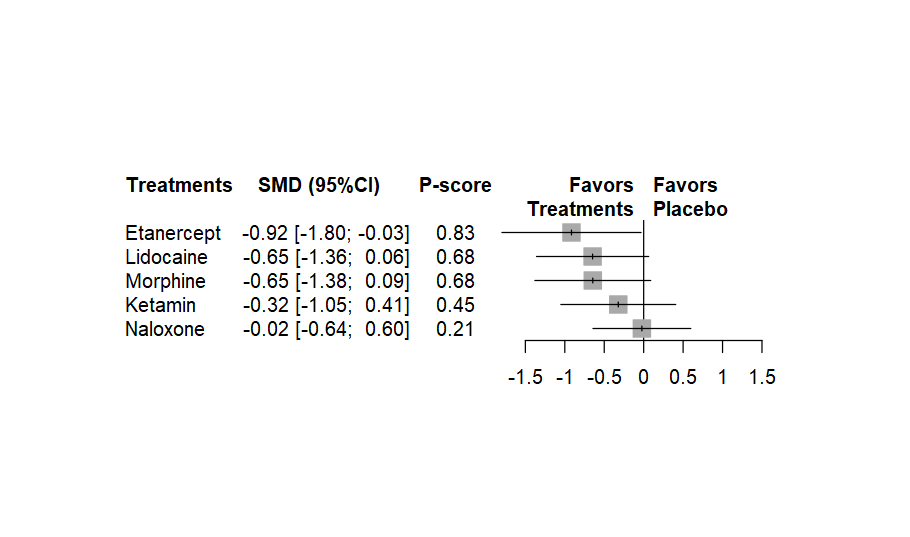


The analysis included trials assessing oral pharmacotherapies. The first-half was a netgraph, and the second-half was a forest plot showing the effect size of each pharmacotherapy against placebo and the P-score.

1. **Study design**
   1. **Cross-over design**


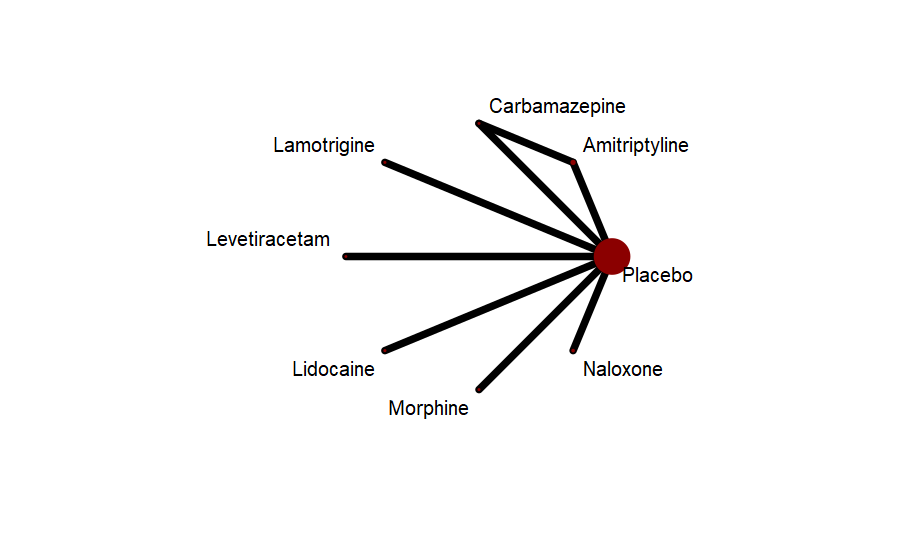


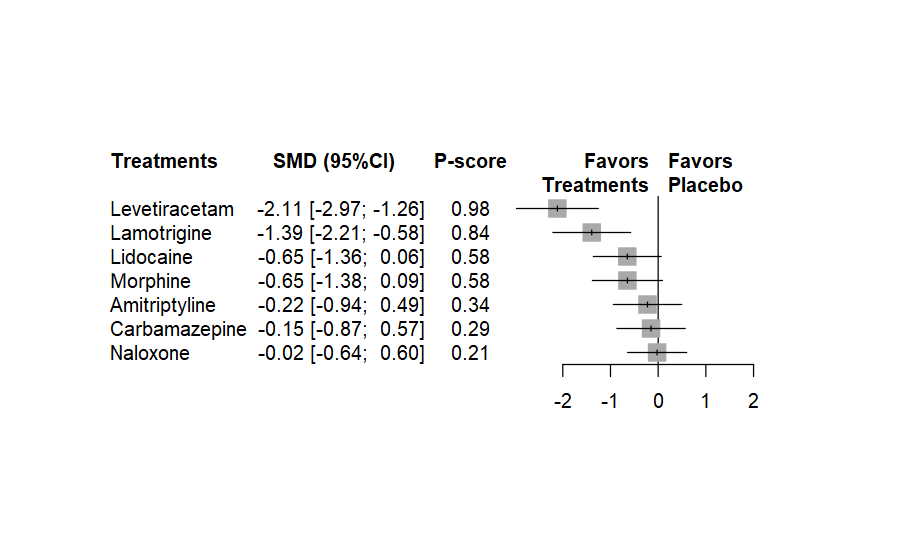


The analysis included trials assessing oral pharmacotherapies. The first-half was a netgraph, and the second-half was a forest plot showing the effect size of each pharmacotherapy against placebo and the P-score.

- 1. **Parallel design**


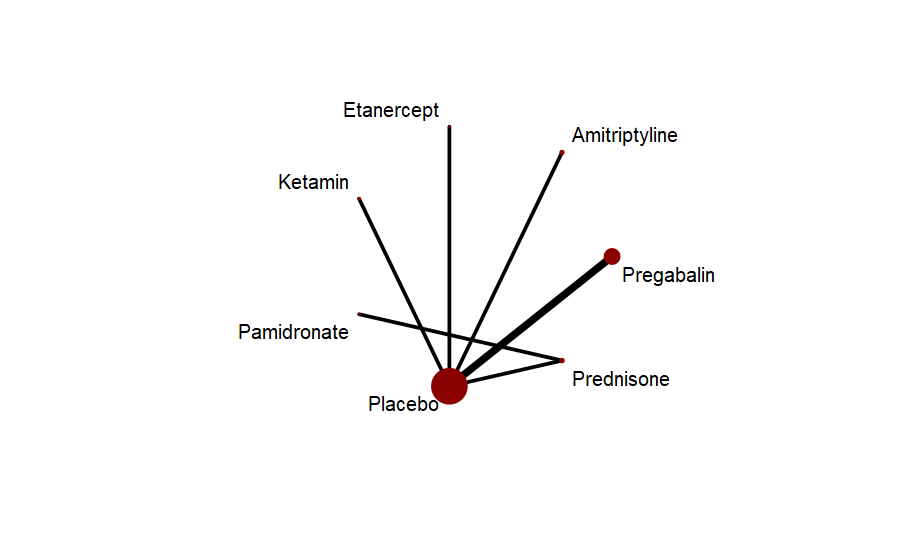


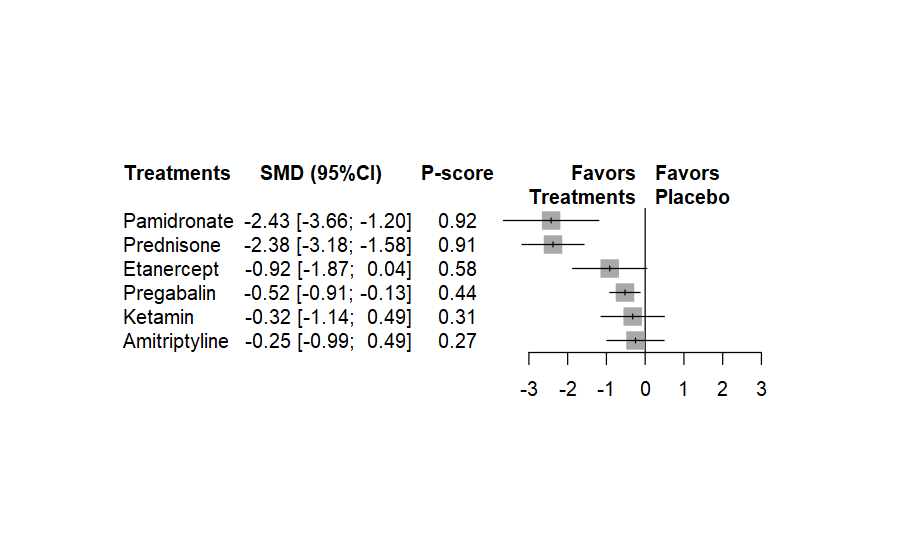


The analysis included trials assessing oral pharmacotherapies. The first-half was a netgraph, and the second-half was a forest plot showing the effect size of each pharmacotherapy against placebo and the P-score.

1. **The causes of CPSP**
   1. **Stroke**


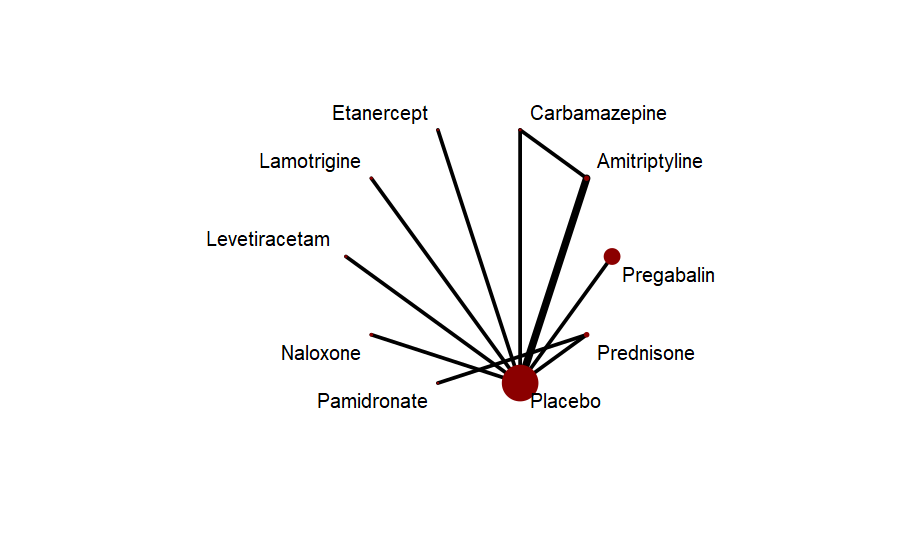


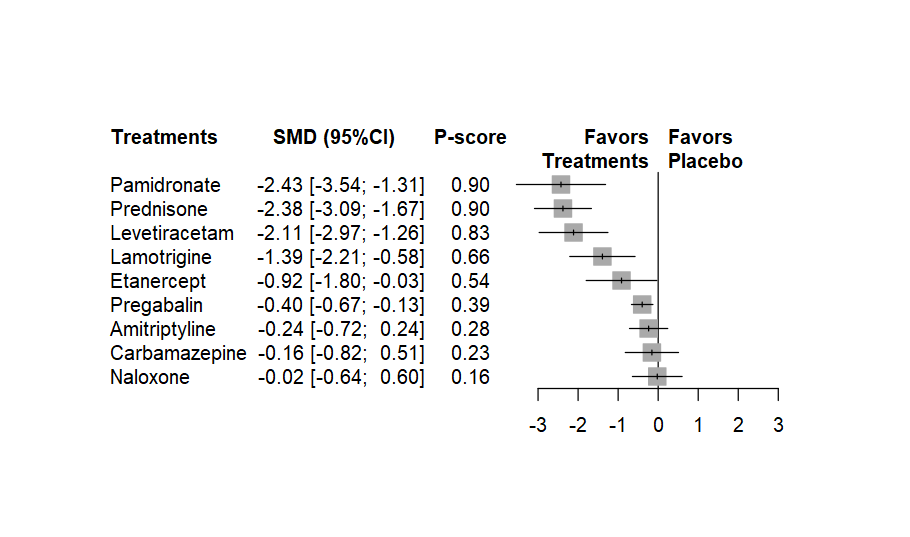


The analysis included trials assessing oral pharmacotherapies. The first-half was a netgraph, and the second-half was a forest plot showing the effect size of each pharmacotherapy against placebo and the P-score.

- 1. **Stroke or spinal cord injury**


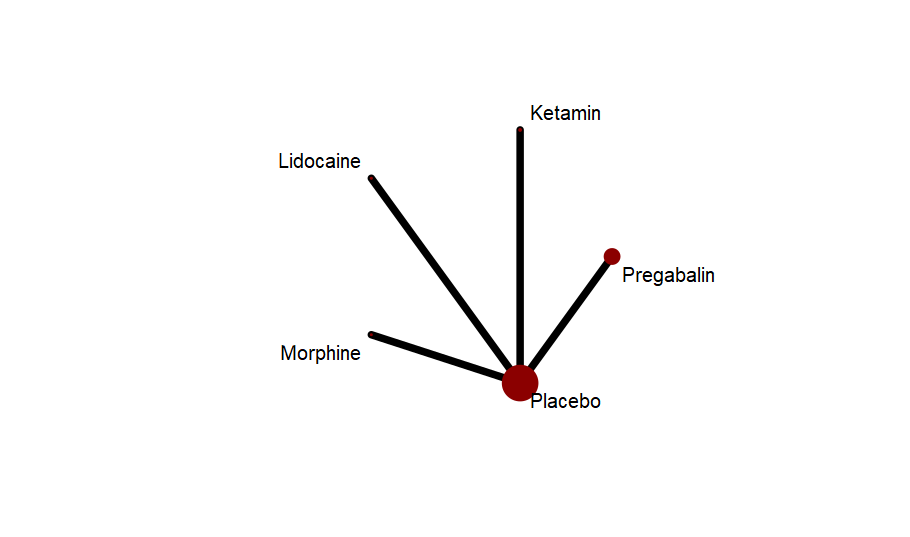


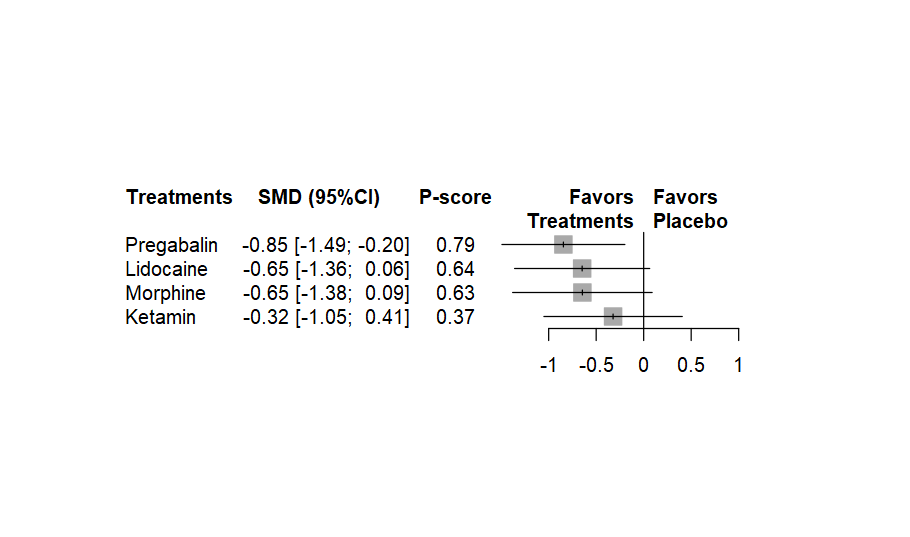


The analysis included trials assessing oral pharmacotherapies. The first-half was a netgraph, and the second-half was a forest plot showing the effect size of each pharmacotherapy against placebo and the P-score.

1. **Measurement methods**
   1. **100-VAS scale**


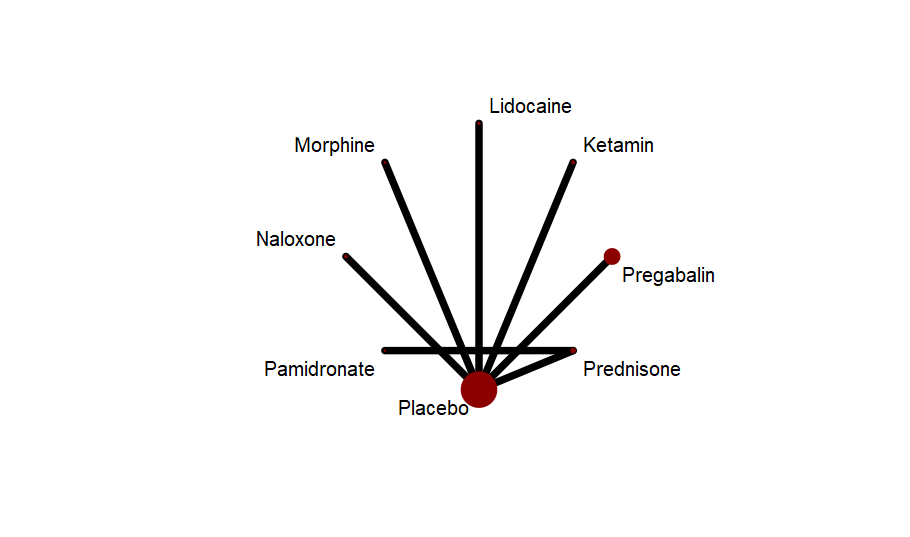


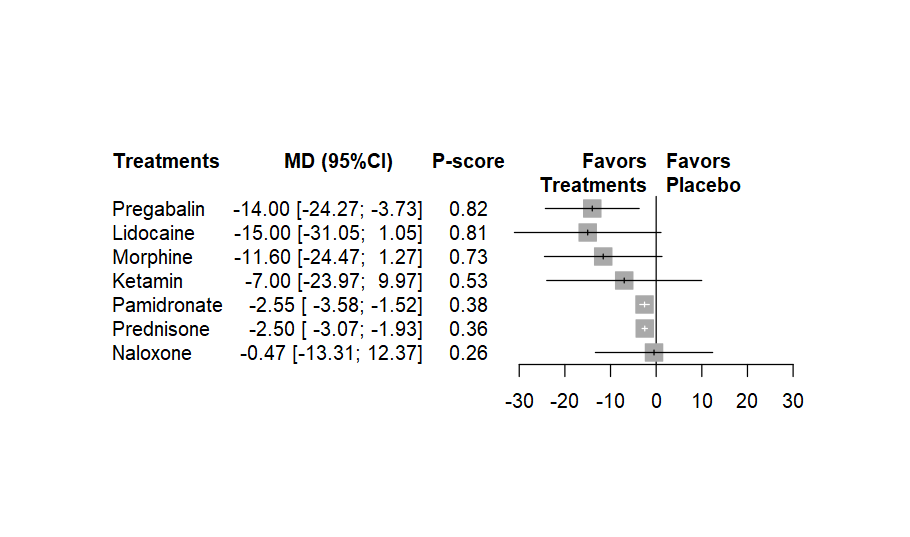


The analysis included trials assessing oral pharmacotherapies. The first-half was a netgraph, and the second-half was a forest plot showing the effect size of each pharmacotherapy against placebo and the P-score.

- 1. **Other scales**


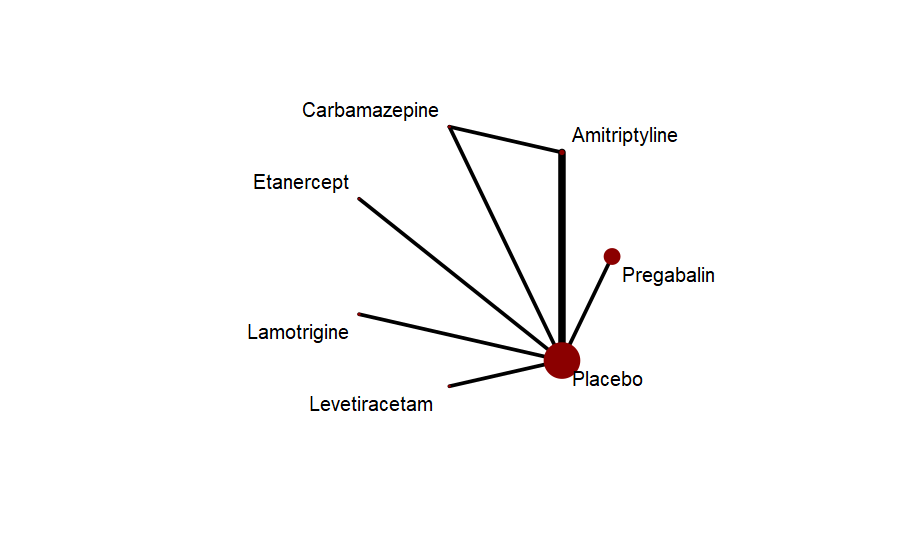


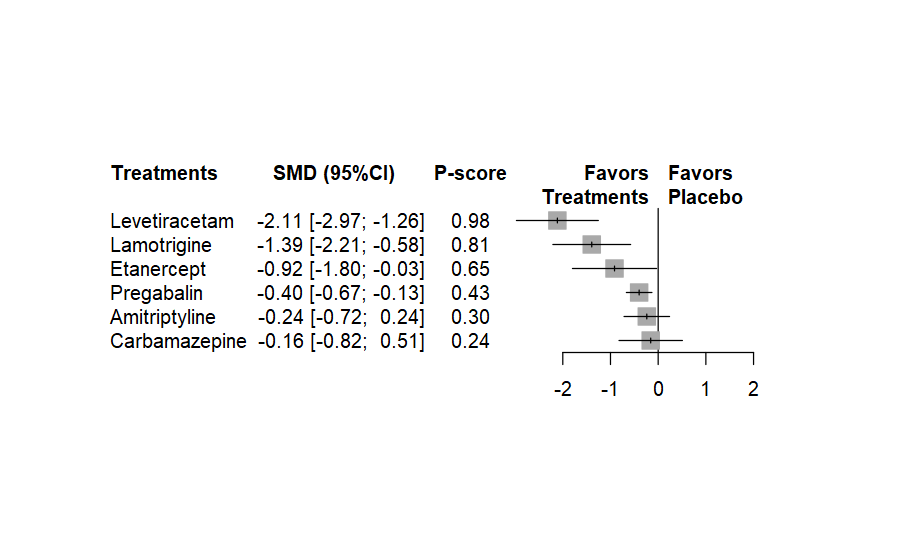


The analysis included trials assessing oral pharmacotherapies. The first-half was a netgraph, and the second-half was a forest plot showing the effect size of each pharmacotherapy against placebo and the P-score.

1. **Treatment duration**
   1. **Short-term duration**


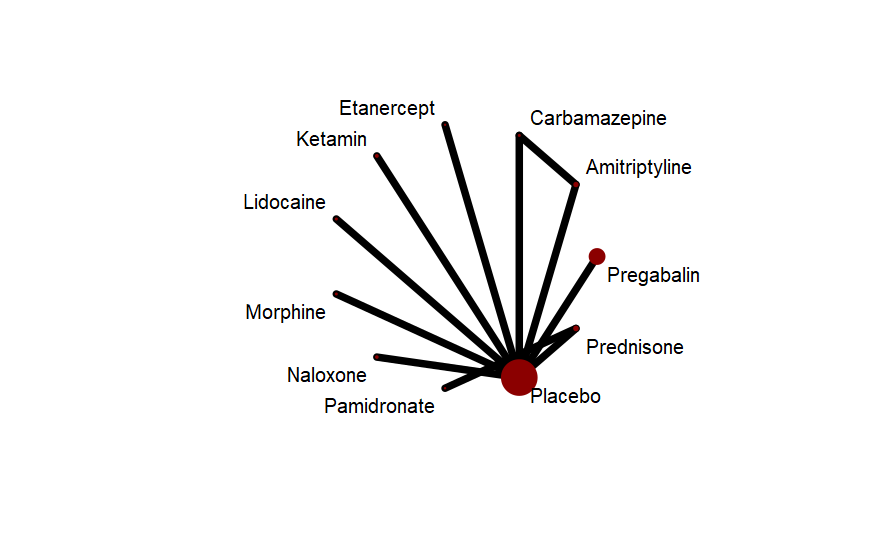


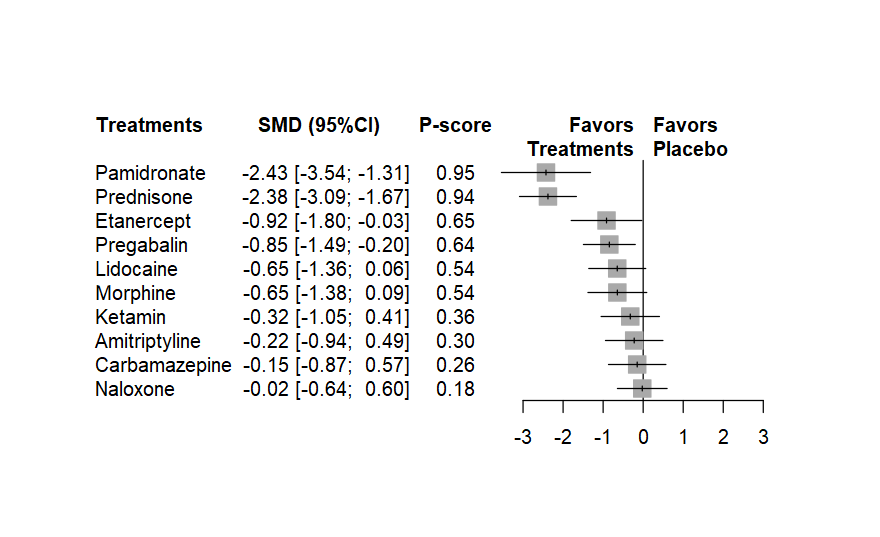


The analysis included trials assessing oral pharmacotherapies. The first-half was a netgraph, and the second-half was a forest plot showing the effect size of each pharmacotherapy against placebo and the P-score.

**5.2 Medium-term duration**


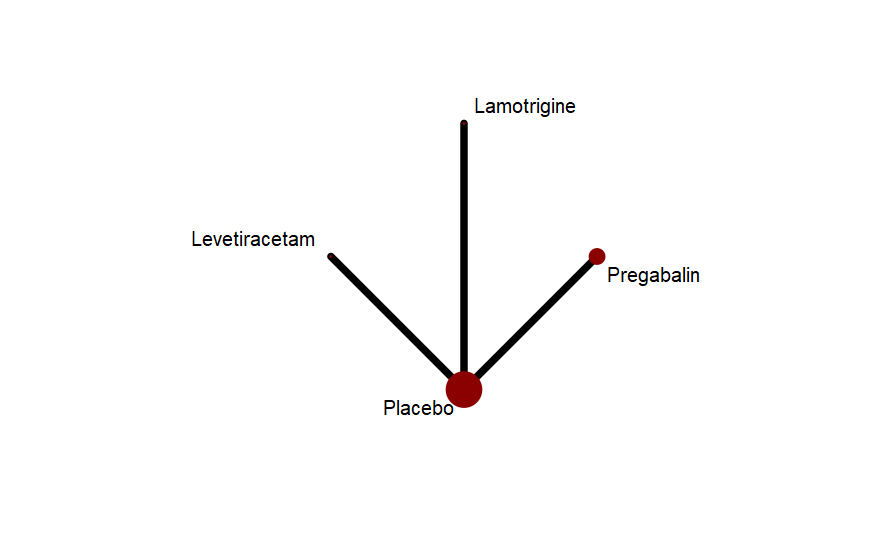


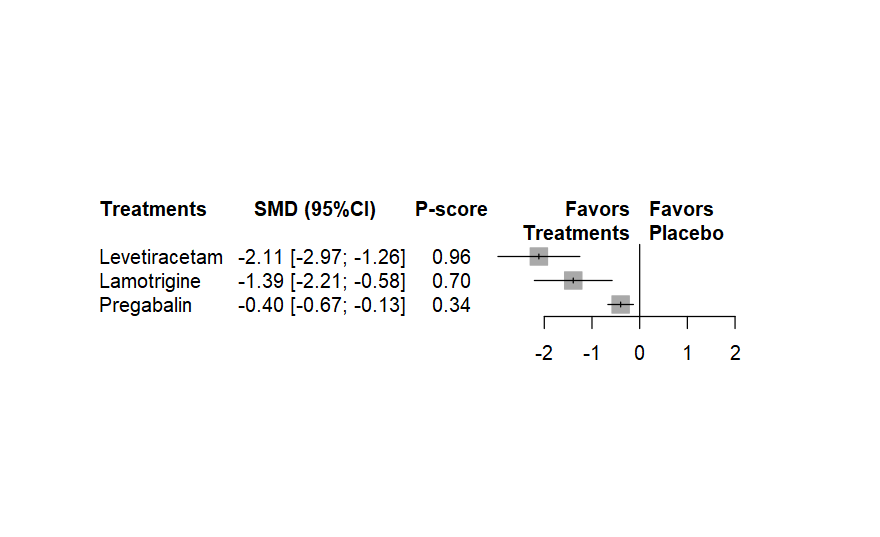


The analysis included trials assessing oral pharmacotherapies. The first-half was a netgraph, and the second-half was a forest plot showing the effect size of each pharmacotherapy against placebo and the P-score.

**5.3 Short-term duration**


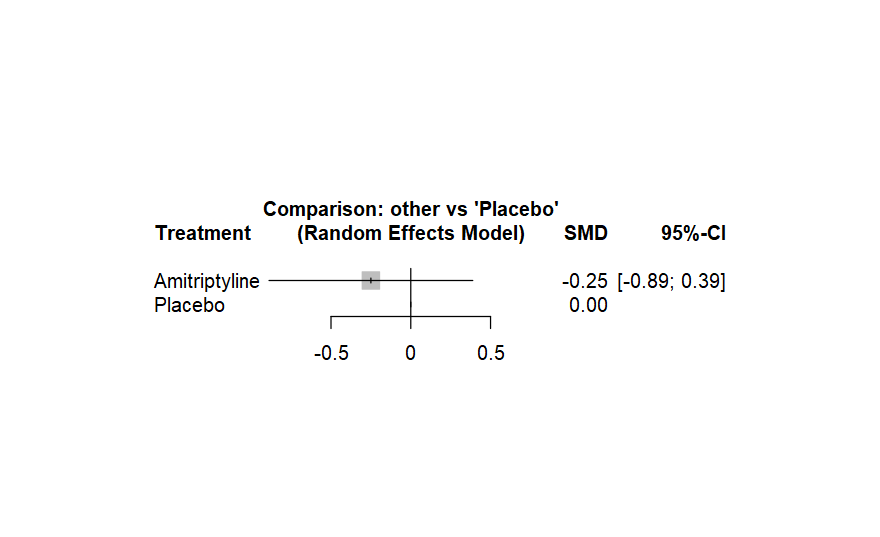


The forest plot showed the effect size of amitriptyline versus placebo.
